# Computer vision-based plants phenotyping: A comprehensive survey

**DOI:** 10.1016/j.isci.2023.108709

**Published:** 2023-12-12

**Authors:** Talha Meraj, Muhammad Imran Sharif, Mudassar Raza, Amerah Alabrah, Seifedine Kadry, Amir H. Gandomi

**Affiliations:** 1 Department of Computer Science, COMSATS University Islamabad Wah Campus, Wah Cantt 47040, Pakistan; 2 Department of Information Systems, College of Computer and Information Sciences, King Saud University, Riyadh 11451, Saudi Arabia; 3 Department of Applied Data Science, Noroff University College, Kristiansand, Norway; 4 MEU Research Unit, Middle East University, Amman 11831, Jordan; 5 Department of Electrical and Computer Engineering, Lebanese American University, Byblos, Lebanon; 6 Faculty of Engineering Information Technology, University of Technology Sydney, Ultimo, NSW 2007, Australia; 7 University Research and Innovation Center (EKIK), Óbuda University, 1034 Budapest, Hungary

**Keywords:** Phenotyping, Plant Biology, Machine learning

## Abstract

The increasing demand for food production due to the growing population is raising the need for more food-productive environments for plants. The genetic behavior of plant traits remains different in different growing environments. However, it is tedious and impossible to look after the individual plant component traits manually. Plant breeders need computer vision-based plant monitoring systems to analyze different plants' productivity and environmental suitability. It leads to performing feasible quantitative analysis, geometric analysis, and yield rate analysis of the plants. Many of the data collection methods have been used by plant breeders according to their needs. In the presented review, most of them are discussed with their corresponding challenges and limitations. Furthermore, the traditional approaches of segmentation and classification of plant phenotyping are also discussed. The data limitation problems and their currently adapted solutions in the computer vision aspect are highlighted, which somehow solve the problem but are not genuine. The available datasets and current issues are enlightened. The presented study covers the plants phenotyping problems, suggested solutions, and current challenges from data collection to classification steps.

## Graphical abstract

**Figure:**
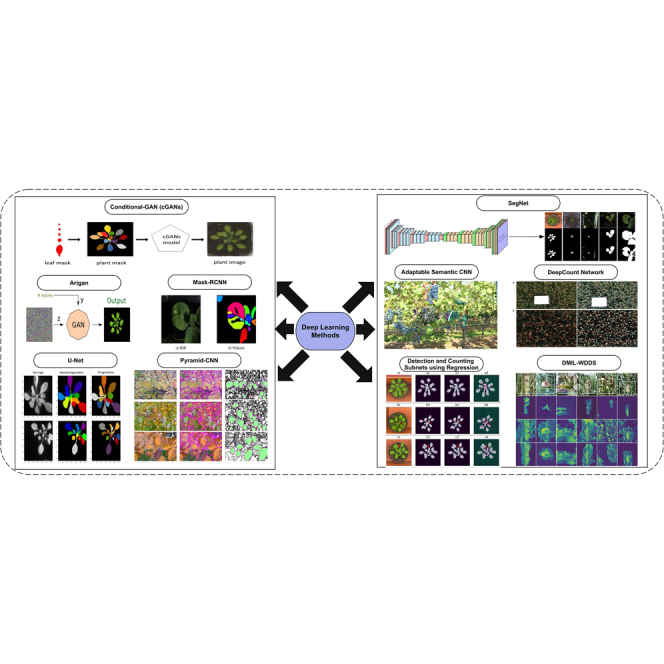

## Introduction

The increasing population of the world potentially raises food production needs. It becomes a grand challenge to preserve the food growth and production environment. It is estimated that the food production needs will dramatically rise to double by 2050. Making agricultural beans more food-productive and environmentally sustainable is essential to meet the projected needs.^1^ Agricultural beans are the food seeds that grow up to make animal and human food. However, in terms of environmental sustainability, arable land is continuously showing huge usability deficits due to an increase in populated urban areas. The arable land can be used for food production and increase the revenue of that particular country.^2^ In this way, the plant’s production can be improved, ultimately fulfilling the ecosystem environment’s food and other needs. The rapidly changing environment affects plants’ growth rate. Plant phenotyping is a term used to understand plants’ anatomical, physiological, ontological, and other chemical properties.^3^ Johannsen first introduced this term in the 1960s. He collected seeds of different types and analyzed that seed sizes behave differently in different environments.^4^

The phenotyping term is not only used for plant traits but also can be used for microbes, animals, and fungi.^5^ Plant phenotyping is a science that can be used for growing development in phenotyping technologies. It is associated with the agronomy and ecophysiology of plant genomics, which is necessary to understand the complex structure of traits. The genotypes and phenotypes make it easier to understand the genetic trait structures that are helpful for functional understanding in different plants.^6^ To select a superior environment for plant growth, the plant breeders still conduct qualitative and quantitative experiments on plants. These quantitative measurements include many properties of traits like plants’ metabolic pathways,^7^ the variety of different proteins,^8^ and the living environment of plants that directly affects plant growth. It is not as simple as the linear analysis of some genotypes. Phenotyping is a complex, challenging task due to thousands of properties of genotypes.^5^ These properties include genetic variation, environmental interactions, multifactorial traits, etc. Therefore, by analyzing these complex properties, a relationship between phenotypes and genotypes is unraveled, contributing to the advancement of medicine, genetics, and the biological fields. In the past, plant breeders did phenotypic measurements in costly, laborious, and time-consuming processes. Also, the manual measurements are not so reliable and are not precise. Therefore, automatic and accurate phenotypic measurements are needed in current times.^9^ However, many applications of phenotyping using computer vision are in use, and some significant uses are discussed in the following sections.

### Remote sensing-based plant phenotyping

The use of robotics with computer vision-based methods also provides ground-level plant phenotyping screening. Similarly, aerial view drones, robot like BoniRob,^10^ and other satellite imaging-based plant phenotyping detection play an important role in remote sensing-based data collection. Crops and weeds can be recognized and classified using this technology.^11^ In the old days, before computer vision-based methods, these measurements were laborious, time-consuming, and imperfect as well.^12^ Moreover, using computer vision methods, crop components can be segmented, pest detection can be applied, and many other crop diseases can be recognized.^13^

Furthermore, remote sensing involves using sensors to capture data regarding the electromagnetic spectrum, including visible, thermal, and hyperspectral ranges. One commonly used remote sensing technique is hyperspectral imaging technique,^14^ which allows for analysis of the leaf pigment, water status, disease detection, etc. Similarly, light detection and range (LiDAR) imaging^15^ measures plant heights, canopy structure, and 3D modeling.

### Aircraft vehicles-based plant phenotyping

Farmers in many developed countries like Thailand and Germany use unmanned aircraft^16^ for field monitoring, crop yield calculations, and pest detection. The images taken from aircraft are of high resolution with spatial information, are low in cost, and are easily operational. The aircraft provides much help for crop and soil monitoring using machine learning (ML) techniques^17^ to recognize the best soil for a specific crop type with water and other sanitary conditions estimation. Furthermore, aircraft-based plant phenotyping has different significant benefits, such as providing large-scale coverage, assisting in data collection, and reaching unreachable places on foot.

**Table:** List of abbreviations

H.I	harvesting index
ML	machine learning
DL	deep learning
GIS	geographical information system
GPS	global positioning service
PET	position emission tomography
MRI	magnetic resonance imaging
W.R.T	with respect to
SBD	symmetric best Dice
BD	best Dice
DiC	difference in count
DBSCAN	density-based spatial clustering
ACT	ant colony optimization
LSC	leaf segmentation challenge
MSR	multi-scale regression
ANFIS	adaptive neuro-fuzzy inference system
GAN	generative adversarial network

The abbreviations used in this survey are described.

### Satellite imaging-based plant phenotyping

The increasing demand for food makes each government need to support their agricultural system. Satellite and the images collected by some means are used for biomass estimation and other crop yields.^18^ The pH of soil, soil moisture, and other soil measurement-based calculations can be estimated using satellite imageries.^19^ Furthermore, regular and consistent monitoring for a large-scale area could be monitored, and a historical analysis could be performed. Geographical information systems (GISs) often use these images to get more promising results and precision. Many satellite image-providing institutes offer their services, whereas the most common are NASA, Geocento,^20^ Google Earth Engine, and many more. Similarly, forest monitoring in many countries also utilizes satellite imagery to monitor forest climate change.^21^

**Figure 1:**
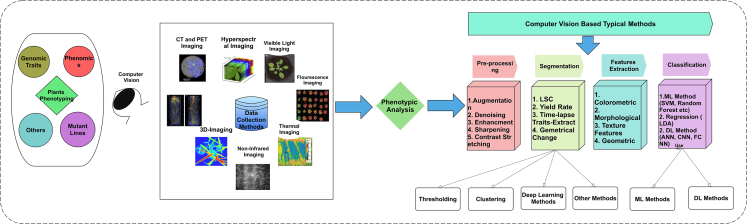
Systematic diagram of the presented survey with basic image processing steps of recognition including image collection methods

From all the previous discussion on plant phenotyping-computer vision applications, we conclude that computer vision assists biologists with plant phenotyping solutions to conduct monitoring and improvements. However, the image collection methods and image processing-assisted solutions face different problems and give solutions to them, which are discussed in this review. Finally, the system diagram of the presented study is shown in Figure 1, where it illustrates all the basic steps discussed in the presented manuscript. In Figure 1, data collection methods section contain different modalities based samples taken from different studies^.^^22^^,^^23^^,^^24^^,^^25^^,^^26^^,^^27^^,^^28^^,^^29^

### Comparison with existing surveys

This survey covers most data acquisition techniques with pros and cons, whereas existing surveys are topic specific regarding technologies or methods. Furthermore, the presented survey covers data collection methods, segmentation, and classification techniques. It also discussed and observed the challenges and problems in the current era using computer vision-assisted methods. The short comparison with previous surveys is shown in Table 1.

**Table 1:** Comparison with existing surveys

Phases	Our survey	Li et al.^30^	Chandra et al.^18^	Mochida et al.^31^	Li et al.^32^
Imaging techniques and problems	✓				✓
Pre-processing	✓		✓	✓	
Segmentation	✓	✓	✓	✓	
Features extraction	✓			✓	
ML classification	✓			✓	
DL classification	✓	✓	✓		

Furthermore, the presented survey covers data collection methods, segmentation, and classification techniques. It also discussed and observed the challenges and problems in the current era using computer vision-assisted methods. The short comparison with previous surveys is shown in Table 1.

### Contributions of this survey

The existing surveys, although much appreciated, are regarding discussion on plant phenotyping techniques and methods. However, a single report on major modalities of plant phenotyping with corresponding challenges and solutions is not discussed.

Therefore, we present a review of plant phenotyping covering the major and traditional methods including:

1. a detailed discussion of data acquisition methods against different species and the acquired challenges that need to be solved;
2. an insightful discussion on challenges that need to be met via segmentation, leaf counting, and classification; and
3. a detailed discussion on previously applied ML and deep learning (DL) approaches for segmentation and classification with their shortcomings and strengths

The remaining review contains these sections: computer vision-based plant phenotyping, pre-processing, segmentation methods with their different approaches, feature extraction methods from multiple aspects, and classification methods using ML and DL with their corresponding results. The last sections contain current challenges and the availabilities of a few famous datasets.

## Computer vision-based plant phenotyping

The plant’s phenotyping-based experimental designs were made using computer vision methods for the quantitative measurements. It analyzes the gene-environment, plant-growing infrastructures, substrate handling, and other monitoring-based installations. To make standard protocols for plant monitoring, the imaging sensor needs precise imaging data collection and processing. The quantitative measures of phenotype data need metadata for the evaluation of results. By evaluation, we can control the growing environment from the field, in a greenhouse, or in plant growth chambers. Many plant imaging techniques can be used for explained purposes, from which some available datasets are discussed in the coming sections.

### Image collection tools and challenges

Different imaging data can be categorized into two major types of plants: below-ground and above-ground plant-based imaging. These types are discussed individually with their current challenges, mode of use, limitations, desired outcomes, and the targeted species with their concerning components.

#### Plant above-ground organ phenotyping

**Figure 2:**
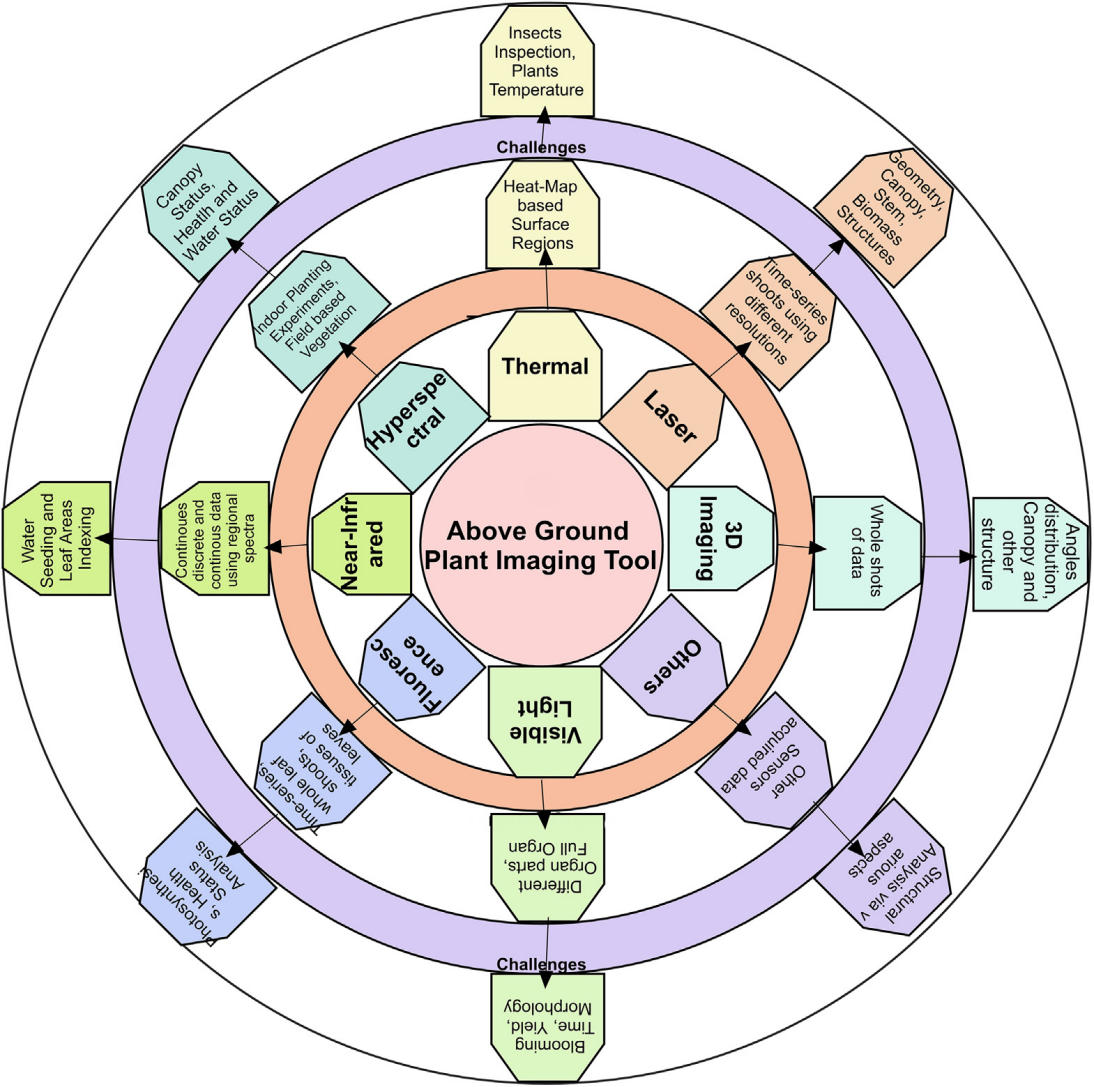
The challenges acquired on different organs of plants (above-ground) using machine learning

Plants grow from the soil and appear in the ground, whereas their roots remain below ground. For above ground, many image collection methods are used nowadays, such as thermal, hyperspectral, visible light, laser, fluorescence, near-infrared, and 3D imaging. To collect data using each technology, there are certain limitations and challenges such as occlusion fact, light effects, and the growing variation of different plants in different growing environments. A few methods presented by previous studies using particular species organs with their reporting quantitative metrics are shown in Figure 2.

Among above-ground image collection modalities, thermal imaging is used for biotic status information, surface temperature, and water stress analysis. It is also limited due to variations in the atmospheric behavior of plants. Some species, such as grapevine,^24^ barley,^33^ maize,^34^ wheat,^35^ and rice,^24^ are analyzed using thermal imaging as a basic image acquisition step. Studies propose metrics that inspect plants and measure their structures' temperature. However, differentiating between soil and plant temperature is still challenging, even using thermal sensors. Likewise, hyperspectral imaging techniques are used to get water leaf area measurements, leaf growth, and other health statuses of multiple growth stages. It is limited due to its cost, complexity, and big interpretation problems.

Some hyperspectral imaging data are used to analyze certain species such as *Arabidopsis*,^36^ rice, triticale,^37^ and wheat, which output the canopy status. In contrast, a few of the water-based health statuses are also analyzed. However, the pigments concatenation, plant stress, and water-controlling environments are acquired challenges using this imaging tool. The sunlight influences the image collection angle for canopy structural analysis. Therefore, it needs to change frequently, even in one round.

However, visible light imaging has many limitations, such as it returns only physiological information about plants in a controlled environment. It also limits the calibration process for spectral information collection; the sunlight under shadow may cause over-exposure to process the images further. In visible light imaging, the production analysis uses area-based yield, the growth rate uses morphology, and the time slot is calculated. Likewise, plant blooming is also noticed in various species using their images, such as *Arabidopsis thaliana*, bean, barley, citrus fruits,^38^ maize,^39^ rice, and *Medicago truncatula*.^40^ A certain range of specific scanners is required for the laser-based image collection method. The embedding with GPS (global positioning service) acquired georeferencing. Further, the particular illumination is also a problem in a controlled environment.

Similarly, when using fluorescence imaging, a pre-acclimation state is required for complicated analysis of biotic and abiotic measurements that are difficult to achieve using visible light imaging in a field environment. The canopy structure, angle-based leaves, and stem-based architect analysis can be done using computer vision-based techniques via laser imaging. Hence, some of the geometric analysis, canopy, and biomass structure analysis are performed on species such as barely, wheat, sugar beet,^41^ triticale,^37^ and soybean. Fluorescence imaging can be used as emitted light from different organs of plants and red-region map analysis wherein the output of computer vision automation, the health status of leaves, photosynthesis, and photochemical analysis can be performed on various species such as *Arabidopsis*, bean, barley, chicory plant,^42^ tomato, sugar, and wheat. The near-infrared imaging can be used as night vision analysis as well. The continuous and discrete data using these sensors are created where they contains region-based spectra data. Time analysis with single shoot and multi-shoots of canopies assessments can be performed wherein output, the water controlling contents measures, and the seeds indexes concerning leaf areas can be analyzed. Near-infrared-based species analysis is performed, including barley, maize, rice, soybean, and wheat.

From the earlier discussion on different types of image collection methods, it is concluded that many analyses are performed using above-ground imaging techniques for different kinds of imaging tools for other species. Growth, structural analysis, time-based single and multiple shoots, stress analysis, surface analysis, health status, photosynthesis, and much more analysis are performed, leading to control and effectively increasing plant production.

#### Plant below-ground organ phenotyping

Other techniques like positron emission tomography (PET), magnetic resonance imaging (MRI), and X-ray imaging are used for below-ground plant-growing environment and structure analysis. In X-ray technology, computer-processed images have been taken by scanning the objects and specific parts to take an inner 3D view of that particular object. It provides us with volumetric data when used in plants’ environments, and then a soil analysis can be performed that can help us measure root systems. MRI modality assists in analyzing soil organs of the plant root system for water distribution or in estimating the water outflow and other quantitative^32^ measures. The water content analysis using 3D morphological patterns can be detected using MRI whereas in some species such as bean, sugar beet, *Beta vulgaris*, and *Hordeum spontaneum*^26^ MRI-based analysis was performed. Likewise, soil and water measurements can also take place using MRI images.

Similarly, PET is also used to get information on plants' functional processes using nuclear or gamma rays. It is used to get images via a pair of nuclear (gamma) rays injected via a PET sensor. It can be used as an individual or also can be used by fusing with MRI technology. However, using these 3D data, we can analyze the functional position of water in plant organs.

Furthermore, the velocity of water movement can be predicted using PET imaging. After acquiring plant data from different aspects of tools and technologies, the next step is to use the computer vision cycle of pre-processing, segmentation, feature extraction, and classification, which will be discussed in the coming sections. The transport analysis uses emitted signals, whereas sector-wise analysis can also be performed using these images. However, many species, such as *Beta vulgaris* and *Hordeum spontaneum*,^26^ are being analyzed, and velocity measurement, transportation, and sectorality are also used to be measured via computer-vision intelligent methods. CT scans are used in many medical and natural imaging analyses, whereas plant phenotyping can provide slices of voxels and plant tissues. Grain quality measurement and 3D structural analysis can also be analyzed. However, these analyses are performed for a few species, such as wheat and rice.^28^ Including all types of image collection tools and technologies discussed earlier, certain species are frequently used. We can analyze the demand for and use of computer vision-based monitoring systems specifically using these species.

### Pre-processing

Pre-processing is an important step in image processing as it enhances the region of interest, ultimately leading to a more precise segmentation or classification. However, it also helps expose tiny grains and spiked particles of plants. Many other data augmentation techniques like cropping, rotation, and scale in variances are used, which increase variations to make patterns much more generalized and robust.^31^ Many noise reduction and enhancement methods are proposed in the image processing domain that could be used in plant imaging noise removal. Enhances such as salt and pepper noise are removed using a fuzzy operator and morphological operator-based method. In this method,^43^ erosion and dilation are used. In contrast, noise is removed using a morphological dual operator, and the peak signal-to-noise ratio (PSNR) metric is used to evaluate the proposed method to validate its performance over other methods. This salt and pepper noise was also removed using a non-linear filter on images, where it was built using hybridization of multiple methods.^44^

A noise reduction approach is proposed for monochromatic images. It is a two-phase noise reduction approach as it first detects noise and then removes it using the adaptive filtering method. The proposed study PSNR values show the method’s robustness in removing the noise from image.^45^

The applied methods of pre-processing in image processing are used to enhance the region of interest by removing noise, enhancing their contrast level, etc. Therefore, if some noise and problems are left while collecting plant phenotyping images, they could be removed by applying these methods.

### Segmentation

Segmenting and analyzing the parts of plants are challenging tasks as organs move and rotate, whereas size and shapes also vary with respect to time. To measure or count the area of organs, estimate the length and width of plant components, detect the degree of sloping and azimuth angle, look into the characteristics of leaf vein, growth rate, and dynamic motion detection, and carry out many more tasks, it is necessary to isolate them precisely. The segmentation of fruit crop diseases using DL techniques and other features was also proposed previously.

#### Segmentation evaluation measures in plant phenotyping

There are several evaluation measures of segmented components in which the Dice score is the most basic and essential metric. It calculates the pixel-wise area of ground truth vs. predicted. The Dice score is modified with multi-label objects in the case of a plant to get the average of each predicted part of the plant as compared to the overall object. Therefore, the modified Dice score is a symmetric best Dice (SBD) score. Both have been shown in Equations 1 and 2.

(Equation 1) $$Dice=\frac{mgtmpt}{\left|mgt\right|+\left|mpt\right|}$$

Dice score is calculated as a ratio of the overlapping area between ground-truth mask *m*^*gt*^ and predicted mask *m*^*pr*^ with the union of *m*^*gt*^ and *m*^*pr*^ whereas the overlapping area is multiplied by 2. This Dice score is then updated as plants have many components, not only one. Therefore, it is necessary to calculate the Dice score accordingly, considering all leaf components’ maximum Dice-yielding score and calculating it by getting the average, as shown in Equation 2.

In Equation 2, *l* denotes the leaf area, whereas *l*^*A*^ and *l*^*B*^ are the sets of leaf components.

(Equation 2) $$BD\left(l^{A},l^{B}\right)=\frac{1}{P}\sum_{x=1}^{P}\begin{array}{c} \max\\ 1\leq y\leq Q \end{array}\frac{2|l_{x}^{A}\cap l_{y}^{B}|}{|l_{x}^{A}|+|l_{Y}^{B}|}$$

Here (1 *≤ x ≤ P*) and (1 *≤ y ≤ Q*) are the ranges for leaf segments (*l*^*A*^ and *l*^*B*^). These best Dice scores are used by SBD to get the minimum from best Dice of predicted (*M*^*Pr*^) and ground truth (*M*^*gt*^) labeled mask with ground truth (*M*^*gt*^) and predicted masks (*M*^*Pr*^) as shown in Equation 3.

(Equation 3) SBD(Mpr,Mgt)=Xmin[BD(Mpr,Mgt),BD(Mgt,Mpr)]

For the leaf count challenge, the leaves after segmentation are counted mostly, whereas, in some other techniques, it is counted without segmentation and reported as a difference in count (DiC), as shown in Equation 4.

(Equation 4) $$DiC=\left|{Lpr}_{f.r}\;-{Lgt}_{f.r}\;\right|$$

The leaf count frequency of the predicted (*L*^*pr*^) mask is subtracted from the actual mask (*L*^*gt*^) to get the DiC from the given object of plants. All these measures are given by a collection study on computer vision-based plant phenotyping segmentation.^46^ Many types of image processing, ML, and DL have been used in previous studies for plant subject segmentation, which will be discussed in the coming sections.

#### Threshold-based segmentation

**Figure 3:**
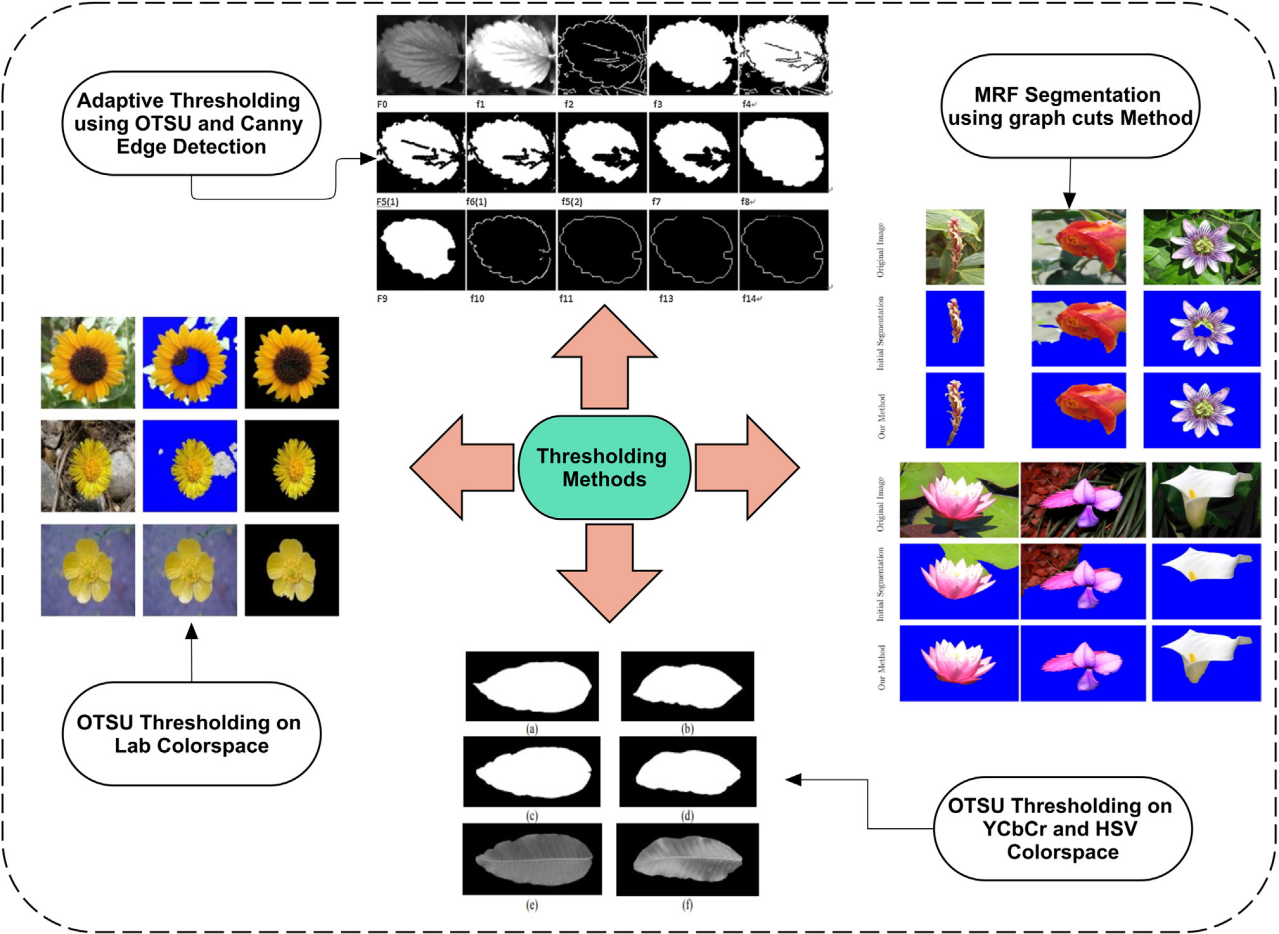
Thresholding-based image segmentation methods

The threshold is the most common and old method in image processing to isolate an object from the image, which is much faster and easier to implement. However, in previous years before 2017, it was mostly used for segmentation, whereas few of their methods are discussed here. Using the Oxford flower color dataset and Lab color space, an OTSU thresholding-based segmentation of flowers is performed. It improves the time efficiency and results compared to a previous study.^47^ It reported results regarding the mean-overlap score using 13 different types of flowers.^48^ The visual results of some threshold techniques taken from cited studies are shown in Figure 3. Different 4 samples taken from previous studies^47^^,^^48^^,^^49^^,^^50^ which used thresholding methods for segmentation of objects.

The jujube leaf image segmentation is adopted using the canny edge detection and OTSU thresholding method, in which an optimization and mapping function is also used to segment the real-life video or images of the jujube plant. Furthermore, the same method is suggested to segment other plant types.^49^ An OTSU method-based thresholding on hue, saturation, value (HSV) and YCbCr color spaces is applied on a mango plant leaf and then compared using precision, recall, and F1-score. It is further suggested that the Cr component is the right color for mango leaf image segmentation.^50^

Another method for mango leaf disease recognition and segmentation is proposed using DL, whereas shape, color, and texture features are extracted from pre-processed images.^51^ Similarly, another study^52^ proposed a fully connected convolutional neural network (CNN) model for mango leaf disease detection. The accuracy reached up to 99.2%. The local contrast haze reduction method is applied to pre-process the images and then fed to the proposed fully connected CNN model. The discussed studies use different imaging methods to apply their manually calculated values for image segmentation. These image segmentation methods are mainly using thresholding techniques and other evaluation metrics.

#### Clustering-based segmentation

A clustering method-based time series analysis on the Panicoid Phenomap-1 public dataset is performed. The stem angles of maize plants are being analyzed in their growth life cycle. A certain behavior of temporal patterns is summarized in three main groups. It is reported that stem angle temporal variations are regulated with the help of genetic variations.^53^ The kiwifruits in day and night images are clustered out to count and segment fruits from the background.^54^ The calyxes line-based adjacent fruits are distinguished and counted. The daytime-based fruit calyxes are detected with 93.7% accuracy, whereas nighttime images with flash are correctly detected with 92% accuracy.

**Figure 4:**
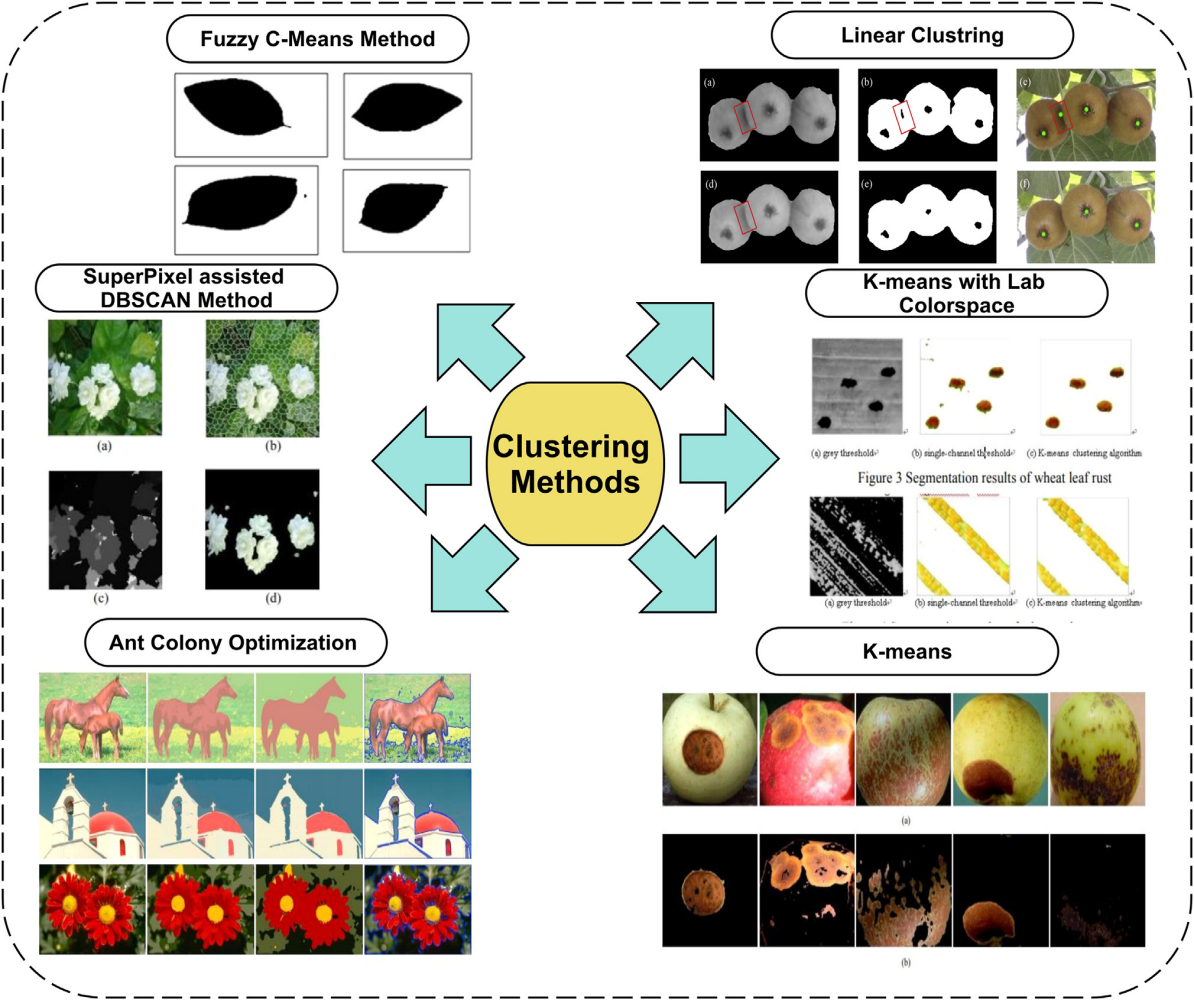
Clustering-based image segmentation methods

To meet the needs of the perfume industry and herbs used in the medical field, it is necessary to calculate the jasmine flowers in the field, which costs more labor. Therefore, the image processing-based segmentation and counting of such challenging tasks are proposed by researchers. The density-based spatial clustering method density-based spatial clustering of applications with noise (DBSCAN) uses neighborhood density information to iteratively cluster and segment the jasmine flowers.^55^ Some of the clustering-based image segmentation results are shown in Figure 4. In Figure 4, some of the clustering method based segmentation results are taken from previous studies.^54^^,^^55^^,^^56^^,^^57^^,^^58^

The defective part segmentation of apple plants using the k-means method is proposed, which uses various steps to make clusters of the defective part. The color features and spatial information cluster pixels at first, and then clustered parts are merged for certain regions. It decreased each pixel cluster calculation time and finally showed that the proposed study is promising to segment out the defected parts.^57^ Disease prediction in plants is necessary to avoid decreasing the yield of that plant. Similarly, wheat production is affected by wheat diseases. Three types of wheat disease, stripe rust, powdery mildew, and leaf rust, are segmented by converting RGB images into Lab color space. The results show that the 90% accuracy of these segmented-out wheat diseases is achieved.^58^ Another method of clustering using the regularized sub-space method is adopted for hyperspectral images.^59^ Three datasets, namely, Indian Pines, University of Pavia, and Salinas, have been used. The widely spread land pictures segment the trees, meadows, bricks, etc. However, the applied method uses a regularized method with a sub-space method that includes spatial information and improves the performance of the sub-space method. It achieved an overall 99% accuracy at the end.

#### DL-based segmentation

It was observed from the literature that DL-based segmentation was primarily adopted after 2016. However, DL-based image segmentation solutions are used in bio-medical, natural, and plants. These solutions are more promising as they use big data for training, making DL solutions more confident than other methods.

**Figure 5:**
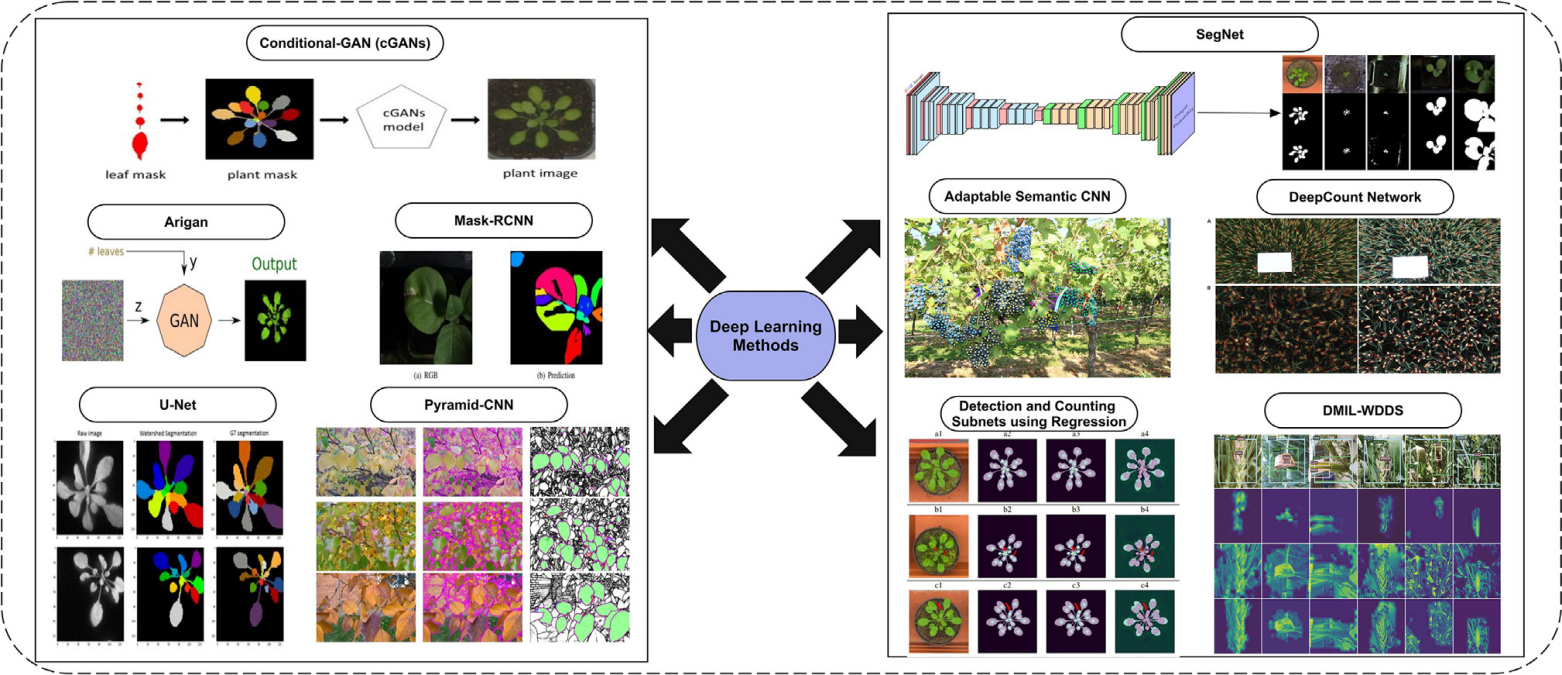
Deep Learning-based image segmentation methods

A study proposed a solution for segmentation and leaf counting challenges using deep deconvolution and convolutional methods. At first, segmentation uses a deep deconvolutional approach, whereas counting is performed using the deep convolutional method. Previously published datasets are used for evaluation purposes, and absolute count difference is reported with a mean of 1.6 and a standard deviation of 2.30.^60^ Some of the DL model-based segmentation results are shown in Figure 5. In Figure 5, few segmentation methods have been applied and visual results are shown that are taken from previous studies.^60^^,^^61^^,^^62^^,^^63^^,^^64^^,^^65^^,^^66^^,^^67^^,^^68^^,^^69^

A custom branch method proposed an instance segmentation solution for wheat disease recognition and localization. It used one public dataset and a self-collected wheat disease dataset. One method, VGGFCNVD-16, and another method, VGG-FCN-S, outperformed the mean accuracy of 97.95% for 1st model and 95.12% for the second model. It also develops a mobile app for this localization purpose.^63^ Similar to this study, which not only gave recognition and localization challenges solutions with a dataset, another study also gave solutions and a dataset with their ground-truth labels. The dataset is named Dense Leaves. A pyramid CNN approach is proposed to detect interior texture. The detected boundaries are then used for estimating the overall shape using a watershed algorithm. The reported results are much more promising for leaves having dense foliage.^64^

To handle limited data challenges, different data augmentation techniques are used. To utilize data augmentation, a combined dataset of synthetic and real augmented images of plant phenotyping is used to propose a deep semantic segmentation network. The proposed study tested on five datasets and reported a Leaf Segmentation Challenge (LSC). It achieved 91% accuracy on the A1-test set from CVPP LSC.^68^ Using the same Computer Vision Problems in Plant Phenotyping (CVPPP) 2017 LSC, two methods are proposed for the counting challenge. The first method did counting using direct regression, whereas the 2nd method predicted leaf centers using a deep convolutional network and then did the counting. Both approaches are named Multi-Scale Regression (MSR) and Detection, Regressor (D + R) approaches. The DiC and absolute DiC (ADiC) are reported by comparing the results of previous studies.^62^ A well-known encoder-decoder-based architect of semantic segmentation, U-Net, is also used for *Arabidopsis* leaf segmentation with the post-processing of the watershed method. It uses three datasets to test it on the proposed method, whereas it achieved 95% and 97% Dice coefficient using synthetic data generation. The RGB real images are converted into fluorescence images to increase data training and testing samples for more promising results.^66^

A deep semantic segmentation approach is used to locate, count, and detect plants. This approach is transferable and adaptable. Moreover, it also proposed that all parameters are learnable for training data from end to end. It also reported that the proposed framework could detect different shapes and sizes of plants. Initially, this approach is applied to grapes dataset.^61^ Another study uses a semantic deep network to segment augmented training data. It also uses the iterative linear clustering method for superpixel test patch generation. These superpixel patches assisted the deep-counted model in quantification. The wheat spikes data are used in this study, whereas data samples are also increased using data augmentation method.^65^ To meet big data training challenges, some adversarial data generation methods have also been proposed by some researchers. A study proposes a rosette image data generator. It produces realistic synthetic data for the CVPPP 2017 dataset. It also used the Ax dataset to increase the efficiency of its results. The Ax dataset contains artificially produced plant images.^67^ A similar approach to data generation using conditional generative adversarial networks is proposed. It also uses a semantic network to report the ADiC and DiC with standard deviation. It claims that the average 16.67% leaf counting error is reduced by using these extensively generated training images to meet the limited annotated scarcity problem in plant phenotyping.^69^

The unmanned aerial vehicles (UAVs)-collected images for yellow rust detection in wheat crops have been present to feed the deep convolutional neural network. This model includes inception-ResNet multiple layers and proposes a wide and deep network. An accuracy of 0.85 has been achieved by this model and suggested that combining the spatial and spectral information results in the improvement of model detection.^70^ The symptom-based disease detection in plants via images could play an important role in the yield of crops. However, the limited annotated data availability reduces the model’s performance. A synthetic image generation of fluorescence images has been proposed to remove this limitation.

Furthermore, a U-Net model is trained to segment the diseased part from the images. The model is tested on the empirical fluorescence dataset. It shows 0.73 precision and 0.79 recall scores when tested on the fluorescence dataset.^71^

#### Other methods-based segmentation

Before 2017, some edge detection, watershed,^72^ and region-based techniques were used for multiple purposes, including leaf segmentation and counting of different plants. The fuzzy numerical morphological operations using edge detection on leaves of tobacco plants are proposed. However, it lacks the continuity of edges and can create a detection risk for adjacent leaves edges.^73^ Similarly, a canny edge detection-based approach is also proposed using the Oxford Flowers dataset. It also has disadvantages in the case of many edges.^74^ Another method based upon canny edge detection but using the orange fruits dataset is proposed. It also has the disadvantage of more edge detection in a single image. In contrast, a comparative analysis is also performed using color- and edge-based segmentation methods on the same plant images.^75^ Automatic segmentation of the same orange images is proposed using shape and color analysis. It also proposed a method to measure the overall yield.^76^ The watershed method is also extensively used to segment and count different plants. It affects a complex algorithm but has efficient results for complex images. Moreover, it works well for images having sharp contrast differences in the objects of image.^77^ For apple images, another method improves the segmentation performance using watershed method.^78^ The different segmentation methods with their different evaluation measure and results are shown in Table 2.

**Table 2:** Different segmentation approaches and their result

References	Year	Method	Species	Dataset	Evaluation measures on subsets			
					A1	A2	A3	Overall
Pape and Klukas^79^	2015	3D histogram, distance map	*Arabidopsis Thaliana*, tobacco	leaf segmentation challenge (LSC) A1 = 128, A2 = 40, A3 = 83	BD = 74.4 FBD = 97.0, ADFL = 2.2, DFL = −1.8	BD = 76.9, FBD = 96.3, ADFL = 1.2, DFL = −1.0(1.5)	BD = 53.3, FBD = 94.1, ADFL = 2.8, DFL = −2.0)	BD = 62.6, FBD = 95.3, ADFL = 2.4, DFL = −1.9
Kumar and Domnic^80^	2017	HSV, histogram quantization	*Arabidopsis Thaliana*, tobacco	LSC, A1 = 128, A2 = 31, A3 = 27	FBD = 93.2, DiC = −0.9, *|*DiC*|* = 2.0	FBD = 94.3, DiC = 1.2, *|*DiC*|* = 3.8	FBD = 81.3, DiC = 0.8, *|*DiC*|* = 2.4	FBD = 91.7, DiC = 0.3, *|*DiC*|* = 2.4,
Sakurai et al.^81^	2018	transfer learning	*Arabidopsis Thaliana*, tobacco	LSC, A1 = 128, A2 = 31, A3 = 27	precision = 0.968 recall = 0.983 F1-measure = 0.975	precision = 0.953 recall = 0.958 F1-measure = 0.953	precision = 0.963 recall = 0.941 F1-measure = 0.948	
Trivedi and Gupta^82^	2020	U-Net	*Arabidopsis Thaliana*, tobacco	LSC, A1 = 128, A2 = 31, A3 = 277, A4 = 624	pixel accuracy = 98.16% IoU = 91.63 Dice = 95.63 MAE = 0.0031	pixel accuracy = 99.26% IoU = 84.34 Dice = 91.21 MAE = 0.0013	pixel accuracy = 98.64% IoU = 72.36 Dice = 79.90 MAE = 0.0045	pixel accuracy = 98.69% IoU = 90.98 Dice = 95.05 MAE = 0.0019
Guo et al.^83^	2021	dual attention guided LeafMask	*Arabidopsis Thaliana*, tobacco	leaf segmentation challenge (LSC) A1, A2, A3, A4, A5	BD (A1) = 92.5	BD (A2) = 89.7	BD (A3) = 91.8, BD (A4) = 89.3, BD (A5) = 90.0,	BD = 90
Tu et al.^84^	2022	improved YOLOV3	*Arabidopsis Thaliana*, tobacco, cauliflower	(ABRC) dataset (images = 500, augmented = 2,000), LSC (A1 = 90, augmented = 1,440)		A1 (agreement = 64 DiC = −0.32 *|*DiC*|* = 0.77 MSE = 0.80)		cauliflower (agreement = 75.11 DiC = −0.11 *|*DiC*|* = 0.48 MSE = 0.31)

The images in RGB are converted into L∗a∗b color space, which is proposed as a good method to discriminate between foreground and background. Furthermore, another method is to separate the leaf center points. The leaf segments are separated by applying the split lines method. This study also uses the LSC dataset using the same subsets but claims that the tobacco class is not segmented out well as compared to the *Arabidopsis*-type of plants. It is also suggested that components of shape adjustment be used to improve the segmentation results.^79^ A watershed method with a stem linking algorithm is used to get leaf count after their segmentation. It uses the CVPPP 2014 dataset of LSC and two subsets, A1 and A2, for segmentation.^85^ Another method using RGB to HSV colorspace conversion and a thresholding method of histogram quantization is used for segmentation. It assists in getting a leaf count. It also uses the same LSC dataset by taking three classes, A1, A2, and A3.^80^

Another study uses the same LSC dataset with an 8:1:1 split ratio of training, testing, and validation datasets. It uses the leaf instance segmentation method using Mask-RCNN to segment and count the leaves. The reported actual evaluation measures are used which are DiC, |DiC|, and SBD.^86^ A two-step approach for leaf segmentation is applied to the LSC dataset using three datasets A1 (*Arabidopsis Thaliana*), A2 (*Arabidopsis Thaliana* variant), and A3 (tobacco). The transfer learning methods are used for segmentation as a fully convolutional network (FCN). The initial learning on a major set of plants is adopted in 1st step, whereas in 2nd step the minor dataset transfer learning adaptation is used using FCN. The precision, recall, and F-measure are reported using various numbers of images and adaptation methods.^81^ A study using various patch size variations by doing customization in CNN architect is proposed, which did edge detection at first.

It uses time-lapse images of plants that are fed into CNN as input. Multiple experiments of binary vs. four-way, patch size variation, and single image vs. patch variants approaches are applied. The CNN and random forest classifiers are used at the end with a patch size of 12.^87^

Researchers have proposed many data augmentation techniques in recent years. One of them used single leaves as data augmentation objects with various angles, and the background was taken as transparent. This data augmentation technique differed from previous approaches because others used synthetic data generation methods. It uses a proposed collage method for data augmentation. It is one of the strong contribution-based segmentation methods, whereas it achieved the mean best Dice score (86.7%) on the A1-A5 dataset.^88^ Another method used the DL method U-Net with the proposed architect to segment the A1-A4 class data. It achieved very good accuracy, Jaccard index, and Dice loss but did not report the SBD, DiC, and |DiC| measures.^82^

From the all aforementioned cited studies on segmentation of leaf segmentation and counting challenges, it is observed that the techniques before 2017 or 2018 were mostly based on thresholding, clustering, histogram quantization, 3D histograms, distance mapping, and other graphical methods. However, in and after 2017–2018, most of the studies used DL methods, whereas more valuable metrics were also included, which improved the performance of each task of plant phenotyping. Some techniques also use traditional feature extraction and classification methods, which are discussed in the coming section.

### Features extraction

Feature extraction techniques are used to extract useful information from images. Classifiers use these feature extraction methods to classify the image data into separable classes. Similarly, many plant species are classified using various feature extractor and descriptor techniques. These features are hand-crafted and use techniques like gradient, intensity, texture, and other geometrical formulation methods. A multi-layer perceptron method using visual hand-crafted features is proposed for wheat grain classification into bread and durum. A total of 21 features are extracted from 12 main features to increase the diversity of distinguishing features. The model was trained on 180-grain inputs, whereas it was only tested on 20.^89^

Another method using the same dataset and classes was proposed using different reproduced nine features from the texture, color, and dimensional domains. However, it claims 99% accuracy is achieved with 100% right detection of wheat grains using an adaptive neuro-fuzzy inference system (ANFIS).^90^ The deep features are the rising good fundamental descriptor to distinguish the objects, whereas the fusion of deep features and selection of fruits is also performed in fruits classification.^91^ Similarly, feature fusion based on deep features and selection is performed using partial least regression (PLS) for crop disease classification.^92^ Seed image recognition is proposed using color and morphological features. This study is performed to analyze the Malva alliance’s systematic positions. The taxonomic genus organization is observed on sections and species level.^93^ The joint features are used for cucumber leaf disease detection. It proposed a robust and dimensionality reduction approach for features extraction.^94^ Another study proposes a global and local texture features approach using histogram-level fusion. It uses scale-invariant feature transform (SIFT) and other features, including mean and standard deviation. These visual features are called a bag of visual features, whereas high-resolution remote sensing images are used for scene classification.^95^

Similarly, the features fusion method on grapes leaf^96^ and cucumber leaf^97^ diseases is applied, including salient, deep, and canonical correlation analysis-based feature extraction methods. A linear discriminant method of stepwise selection is used to classify the species of the genus *Cistus*. Different colorimetric and morphological variables, including mean weight, size, shape, and colors, are used; 137 values or features are used for this purpose.^98^

A new feature named venation, which consists of leaf vein patterns responsible for food and water transport, is proposed. It uses different combinations of venation features, which shows that these features are best compared to outline shape. It claims that DL highlights the many hidden features of leaf images.^99^ The different fruit diseases using novel feature selection approaches are applied using cascaded design^100^ whereas entropy-ranked feature selection approach also applied on segmented fruit diseases images that enhance the performance of classifiers.^101^

### Classification

Classification is performed to identify or recognize the actual species of plant. The taxonomical classification, physiological states, and image analysis-related tasks were performed using various ML and DL methods. ML classification uses previously extracted features or calculated features that assist classifiers. DL methods use images directly as input instances and cover the low-, mid-, and high-level features using their structural layers. However, both of these are discussed in the coming section.

#### ML-based methods

Different classical classification methods are used in ML, in which support vector machine (SVM) is most famously used and performed well compared to many other ML classification techniques. However, many studies were conducted in the plant phenotyping domain to classify the multi or binary class problem to recognize the seeds or traits of plants. Different imagery techniques are used in this aspect, as one study uses laser scanning and airborne spectroscopy data of different plants. Data limitation is reduced to some extent by using two techniques: 499 images of 31 different species, different spectroscopy and laser data features, and feature selection to get important features.

The classification is performed using SVM and random forest classifiers, in which SVM performs better than random forest.^102^ Image classification and regression are performed using pre-trained CNN and Auto-Keras models, and the lodging score is used as a model evaluation criterion. The classification as lodged or not lodged is performed on both strategies, whereas pre-trained CNN got 93.2% and Auto-Keras got a slightly lower accuracy of 92.4%.^103^ To supervise the quality monitoring of plants, a study used statistical modeling via the visual perception of images. The spatial structures are taken under consideration to perform complex statistical modeling. An omnidirectional and multi-scale method of Gaussian filtering is proposed to describe the spatial structure of grains. The classification problem is also solved by proposing the multi-kernel least squares SVM method. The food production quality of rice grains is tested by this method.^104^ Many other studies use other ML classification methods, as discussed in the previous section on feature extraction. However, a summary of different classification methods based on their feature extraction methods and classification methods are shown in Table 3.

**Table 3:** Classification results on plants species using ML and DL approaches

Domain	References	Methods	Species	Results
Machine learning	Sabanci et al.^90^	Color, texture features, ANFIS classification Method	bread, durum	Acc = 99%
	Lo Bianco et al.^93^	Shape, size, texture features and LDA classification method	Malva or Lavatera	Acc = 97.6%
	Zhu et al.^95^	Spectral, SIFT, GLCM, SITI features and LGF-BOVW classification method	UC-Merced data, 21 classes	Acc = 96.960.95%
	Lo Bianco et al.^98^	Colorimetric, texture features and LDA classification method	*Cistus* species and sub-species	Acc = 80.6%
	Sadeghi-Tehran et al.^105^	SIFT, LLC, SPM features and SVM classification method	wheat heading, wheat flowers	Acc = 99.59% Acc = 85.45%
	Sabanci et al.^89^	Color, texture features and ANN classification method	bread, durum	MAE = 9.8106
Deep Learning	Mohanty et al.^106^	Deep features and softmax classifier using CNN	14 Crops, 26 diseases	Acc = 99.35%
	Brahimi et al.^107^	Deep features and softmax classifier using CNN	tomato disease	Acc = 99.18%
	Ghosal et al.^108^	Deep features and softmax classifier using CNN	abiotic, biotic diseases	Acc = 90.3%
	Wang et al.^109^	VGG-16, 19, ResNet-50 and Inception-V3	apple black	Highest Acc of VGG19 = 90.4%
	Ferentinos^110^	Deep features and softmax classifier using CNN	25 plant species	Acc = 99.53%
	Ramcharan et al.^111^	Deep features and softmax classifier using CNN	cassava	Acc = 93%
	Liu et al.^112^	Deep features and softmax classifier using CNN	apple diseases	Acc = 97.62%
	Picon et al.^113^	ResNet-50	wheat crop	Balanced Acc = 84%
	Too et al.^114^	Inception, ResNet, DenseNe, VGG	14 plant species	Best Acc = 99.75%

#### DL-based methods

After 2017, DL techniques found much fame in their use for recognition, segmentation, and object detection in medical, natural, and other fields of the real world. However, recent studies on plant species contributed to species recognition.^115^ One of the studies uses big data of more than 54k images, containing 14 crop data and 26 disease data. The testing data using the holdout validation method is tested on a trained DL model. It achieves a surprising classification accuracy of 99.34%.^106^

Similarly, another big dataset of tomato diseases uses more than 14k images. CNN deep layer-based feature extraction detects or localizes the disease area. It also achieves a good accuracy of 99.18%.^107^ Big data usage in DL is also used in other species of plants to accurately identify the species within the testing data belonging to other scales, positions, and angles. Similarly, a study says that our proposed model is robust to image perturbations and highly eligible for real-time deployment. It uses 25k images of plants’ biotic and abiotic diseases, which were causing plant stress. It achieves 90.3% classification accuracy.^108^

Another study used big data as 87k+ for CNN’s training and testing purposes. It contains 58 different types of plant disease data, whereas, when substituted, it contains 25 species data. However, the proposed model achieved 99.53% classification accuracy.^110^ Some other studies use multiple pre-trained models to train them from scratch. A study uses a small amount of data as compared to previous studies. It fine-tuned VGG-16, 19, ResNet-50, and Inception-V3 models. All models perform well, and it is suggested that they be used by training from scratch, whereas the VGG19 model claims the highest accuracy of 90.4%.^109^ A transfer learning-based CNN model is proposed. It uses various types of plant species for their automatic recognition. It is used to detect cassava disease. However, different class-wise classification results are presented, whereas an overall accuracy of 93% is achieved.^111^

Apple leaf internal identification is proposed using five different categories of them. It uses 13k+ images to make more promising results as compared to previous studies.

The 97.62% accuracy is achieved from the proposed CNN architect. It also suggested using their proposed CNN compared to the AlexNet model by reducing the number of parameters and increasing the convergence rate.^112^ Wheat crop disease detection using wheat-14, 15, and 16 datasets is used, which becomes a total of 8k+ images. Full, leaf-mask, and superpixel approaches using ResNet-50 have been used. Various classification accuracies have been discussed, but total balanced accuracy was increased from 78% to 84%.^113^ A study used a comparison of various fine-tuning CNN models in which 14 species data with their 38 different categories were used. It used VGG, Dense-Net, ResNet, and Inception variants with their different layers. The best accuracy was achieved (99.75%) by Dense-Net by reporting that it takes less time and uses fewer parameters.^114^ We have many cited studies in plant species that use mostly big data and many species. It is also observed that more data usage in DL models increases the classification results whereas ML-based approaches do not use big data; also, their results are inaccurate. DL makes the classification or disease identification tasks more challenging by making big data a prerequisite. However, using many public and private datasets can increase the dataset samples for DL models.

### Challenges and problems

We discussed many new and old research trends on plant phenotyping problems and their provided solutions using computer vision. It concludes that many studies have been proposed previously to solve plant phenotyping-related problems, but there are still many open challenges. Data acquisition is the most important first step in the computer vision domain. While collecting data, many problems cause inappropriate collection, such as the naturally varying environment of plants, winds, illumination factor, spatial varying location of plants traits, and many more. It also concludes that while data are collected using visible light or above-ground data collection methods, the complete representation and view from each aspect are not very promising. The below-ground modalities consume more time to collect and analyze the soil environment, and it is also costly to measure individual plants.

For plant phenotyping-based geometrical and yield production measurement, an appropriate method of image collection is also a challenging task. It has many challenges, such as the range and the time measurement, the resolution power, and zoom-in, and zoom-out at some points. The size, geometrical, and morphological measurements will vary based on these problems. Considering the aforementioned problems, we analyzed that a single aspect of image acquisition may not give promising results. In contrast, if multi-modal techniques such as fluorescence, 3D imaging, and visible light imaging are adopted, results could be improved. Furthermore, it is suggested that low-cost, portable, and high-throughput methods be used to analyze the plants’ traits more quickly and cheaply. It is further concluded that various image collection techniques for different species perform different kinds of plant phenotyping analyses.

However, many problems remain, including growth analysis, structural analysis, time-based single and multiple shoots analysis, stress analysis, surface analysis, health status monitoring, photosynthesis, and many others, which could be improved effectively using the latest computer vision techniques. The more accurate and more confident results of DL methods urged us to create more labeled data. However, some researchers make adversarial methods based on data transformation techniques to create resampled data. Hence, labeled big data creation is still needed for segmentation and classification tasks. The existing studies are species-specific on limited data, whereas some of them used 3D methods of image reconstruction using DL methods. The data limitation can be fulfilled in the future using generative adversarial network (GAN)-based methods or by manual annotation of different plant species. Consequently, it will improve the overall performance of plant phenotyping regarding leaf count, time-lapse traits analysis, and other morphological aspects of plants.

### Datasets

Many public and private datasets and their corresponding metadata are available online. In the following, Table 4 illustrates the acquired challenges to meet them using intelligent ML methods. Furthermore, the most famous and publicly used datasets of different color spaces are also shown.

**Table 4:** Plants phenotyping datasets

Dataset	Color space	URLs
Leaf segmentation challenge (LSC)	RGB	https://www.plant-phenotyping.org/datasets-home
Aberystwyth leaf lvaluation (ALED)	RGB	https://doi.org/10.5281/zenodo.168158
Plant pathology	RGB	https://www.kaggle.com/c/plant-pathology-2020-fgvc7
Plant village	RGB	https://plantvillage.psu.edu/
Setaria shoot dataset	HSV	https://www.quantitative-plant.org/dataset/setaria-shoot
Rice root dataset	X-ray	https://www.quantitative-plant.org/dataset/rice-root-gellan
UNL plant phenotyping	RGB	https://plantvision.unl.edu/dataset
Open plant phenotype database	RGB	https://vision.eng.au.dk/open-plant-phenotyping-database/
Plant-CV	Multi	https://plantcv.danforthcenter.org/pages/data.html
Eschikon plant stress	RGB	https://projects.asl.ethz.ch/data-sets/doku.php?id=2018plantstressphenotyping
CWFID	RGB	http://github.com/cwfid
Proximal sensing data	HD visible light	https://data.nal.usda.gov/dataset/high-throughput-phenotyping-data-proximal-sensing-cart

Many other datasets can be found at the site (https://www.quantitative-plant.org/dataset). It contained both annotated and non-annotated 2D and 3D datasets.

## Conclusion

This review comprehensively studied and discussed the shifting paradigms of plant phenotyping methods using computer vision techniques. The data collection methods using various modalities have been discussed in detail. We also summarize their limitations and feasibility issues. Furthermore, the technical methods using intelligent pre-processing, segmentation, feature extraction, and classification techniques are discussed. Different challenges about benchmark datasets are discussed and solved by previous studies, but still there are open challenges. It is concluded that many DL methods enhance the confidence of the previously achieved results by researchers. However, many methods have been proposed regarding data limitation solutions, including manual annotation using various tools, data augmentation, and adversarial image generation methods. However, the more meaningful evaluation measures of segmentation are used to better interpret and solve quantitative measurements in plant phenotyping. Due to environmental variations, recognizing plant categories and diseases, leaf counting, and many more tasks are still challenging. Much research can be proposed in segmentation methods, data limitation solutions, and more meaningful quantitative measures.

## References

[1] Hunter M.C., Smith R.G., Schipanski M.E., Atwood L.W., Mortensen D.A. (2017). Agriculture in 2050: recalibrating targets for sustainable intensification. Bioscience.

[2] Wu X.D., Guo J.L., Han M.Y., Chen G.Q. (2018). An overview of arable land use for the world economy: From source to sink via the global supply chain. Land Use Pol..

[3] Guo Q., Zhu Z. (2006). Phenotyping of plants. Encycl. Anal. Chem. Appl. Theor. Instrum..

[4] Johannsen W. (1911). The genotype conception of heredity. Am. Nat..

[5] Walter A., Frank L., Hund A. (2015). Plant phenotyping: from bean weighing to image analysis. Plant Methods.

[6] Liu H.-J., Yan J. (2019). Crop genome-wide association study: a harvest of biological relevance. Plant J..

[7] Schauer N., Fernie A.R. (2006). Plant metabolomics: towards biological function and mechanism. Trends Plant Sci..

[8] Schulze W.X., Usadel B. (2010). Quantitation in mass-spectrometry-based proteomics. Annu. Rev. Plant Biol..

[9] Zhao C., Zhang Y., Du J., Guo X., Wen W., Gu S., Wang J., Fan J. (2019). Crop phenomics: Current status and perspectives. Front. Plant Sci..

[10] Ruckelshausen A., Biber P., Michael Dorna, Gremmes H., Klose R., Linz A., Rahe F., Resch R., Thiel M., Trautz D. (2009). Bonirob–an autonomous field robot platform for individual plant phenotyping. Precis. Agric..

[11] Fawakherji M., Ali Y., Bloisi D., Pretto A., Nardi D. (2019). 2019 Third IEEE International Confer- Ence on Robotic Computing (IRC).

[12] Gharde Y., Singh P.K., Dubey R.P., Gupta P.K. (2018). Assessment of yield and economic losses in agriculture due to weeds in India. Crop Protect..

[13] Fuentes A., Yoon S., Kim S.C., Park D.S. (2017). A robust deep-learning-based detector for real-time tomato plant diseases and pests recognition. Sensors.

[14] Sarić R., Nguyen V.D., Burge T., Berkowitz O., Trtílek M., Whelan J., Lewsey M.G., Čustović E. (2022). Applications of hyper-spectral imaging in plant phenotyping. Trends Plant Sci..

[15] Jin S., Sun X., Wu F., Su Y., Li Y., Song S., Xu K., Ma Q., Baret F., Jiang D. (2021). Lidar sheds new light on plant phenomics for plant breeding and management: Recent advances and future prospects. ISPRS J. Photogrammetry Remote Sens..

[16] Primicerio J., Di Gennaro S.F., Fiorillo E., Genesio L., Lugato E., Matese A., Vaccari F.P. (2012). A flexible unmanned aerial vehicle for precision agriculture. Precis. Agric..

[17] Pretto A., Aravecchia S., Burgard W., Chebrolu N., Dornhege C., Falck T., Fleckenstein F., Fontenla A., Imperoli M., Khanna R. (2019). Building an aerial-ground robotics system for precision farming: An adaptable solution. arXiv.

[18] Chandra A.L., Vikas Desai S., Guo W., Balasubramanian V.N. (2020). Computer vision with deep learning for plant phenotyping in agriculture: A survey. arXiv.

[19] Ghazali M.F., Wikantika K., Harto A.B., Kondoh A. (2020). Generating soil salinity, soil moisture, soil ph from satellite imagery and its analysis. Inf. Process. Agric..

[20] Geocento (2021).

[21] ESA Earth Online (2021).

[22] Jansen M., Gilmer F., Biskup B., Nagel K.A., Rascher U., Fischbach A., Briem S., Dreissen G., Tittmann S., Braun S. (2009). Simultaneous phenotyping of leaf growth and chlorophyll fluorescence via GROWSCREEN FLUORO allows detection of stress tolerance in arabidopsis thaliana and other rosette plants. Funct. Plant Biol..

[23] Mishra P., Asaari M.S.M., Herrero-Langreo A., Lohumi S., Diezma B., Scheunders P. (2017). Close range hyperspectral imaging of plants: A review. Biosyst. Eng..

[24] Jones H.G., Serraj R., Loveys B.R., Xiong L., Wheaton A., Price A.H. (2009). Thermal infrared imaging of crop canopies for the remote diagnosis and quantification of plant responses to water stress in the field. Funct. Plant Biol..

[25] Sakamoto T., Shibayama M., Kimura A., Takada E. (2011). As- sessment of digital camera-derived vegetation indices in quantitative monitoring of seasonal rice growth. ISPRS J. Photogrammetry Remote Sens..

[26] Poorter H., Bühler J., van Dusschoten D., Climent J., Postma J.A. (2012). Pot size matters: a meta-analysis of the effects of rooting volume on plant growth. Funct. Plant Biol..

[27] Garbout A., Munkholm L.J., Hansen S.B., Petersen B.M., Munk O.L., Pajor R. (2012). The use of pet/ct scanning technique for 3d visualization and quantification of real-time soil/plant interactions. Plant Soil.

[28] Yang W., Xu X., Duan L., Luo Q., Chen S., Zeng S., Liu Q. (2011). High-throughput measurement of rice tillers using a conveyor equipped with x-ray computed tomography. Rev. Sci. Instrum..

[29] Klose R., Penlington J., Ruckelshausen A. (2009). Usability study of 3d time-of-flight cameras for automatic plant phenotyping. Bornimer Agrartechnische Berichte.

[30] Li Z., Guo R., Li M., Chen Y., Li G. (2020). A review of computer vision technologies for plant phenotyping. Comput. Electron. Agric..

[31] Mochida K., Koda S., Inoue K., Hirayama T., Tanaka S., Nishii R., Melgani F. (2019). Computer vision-based phenotyping for im- provement of plant productivity: a machine learning perspective. GigaScience.

[32] Li L., Zhang Q., Huang D. (2014). A review of imaging techniques for plant phenotyping. Sensors.

[33] Munns R., James R.A., Sirault X.R.R., Furbank R.T., Jones H.G. (2010). New phenotyping methods for screening wheat and barley for beneficial responses to water deficit. J. Exp. Bot..

[34] Araus J.L., Serret M.D., Edmeades G.O. (2012). Phenotyping maize for adaptation to drought. Front. Physiol..

[35] Manickavasagan A., Jayas D.S., White N.D.G. (2008). Thermal imaging to detect infestation by cryptolestes ferrugineus inside wheat kernels. J. Stored Prod. Res..

[36] Matsuda O., Tanaka A., Fujita T., Iba K. (2012). Hyperspectral imaging techniques for rapid identification of arabidopsis mutants with altered leaf pigment status. Plant Cell Physiol..

[37] Busemeyer L., Mentrup D., Möller K., Wunder E., Alheit K., Hahn V., Maurer H.P., Reif J.C., Würschum T., Müller J. (2013). Breedvisiona multi-sensor platform for non-destructive field-based phenotyping in plant breeding. Sensors.

[38] Iqbal Z., Khan M.A., Sharif M., Shah J.H., ur Rehman M.H., Javed K. (2018). An automated detection and classification of citrus plant diseases using image processing techniques: A review. Comput. Electron. Agric..

[39] Nagel K.A., Putz A., Gilmer F., Heinz K., Fischbach A., Pfeifer J., Faget M., Blossfeld S., Ernst M., Dimaki C. (2012). Growscreen-rhizo is a novel phenotyping robot enabling simultaneous measurements of root and shoot growth for plants grown in soil-filled rhizotrons. Funct. Plant Biol..

[40] Dias P.M.B., Brunel-Muguet S., Dürr C., Huguet T., Demilly D., Wagner M.-H., Teulat-Merah B. (2011). Qtl analysis of seed germination and pre-emergence growth at extreme temperatures in medicago truncatula. Theor. Appl. Genet..

[41] Paulus S., Behmann J., Mahlein A.K., Plümer L., Kuhlmann H. (2014). Low-cost 3d systems: suitable tools for plant phenotyping. Sensors.

[42] Lootens P., Devacht S., Baert J., Van Waes J., Van Bockstaele E., Roldán-Ruiz I. (2011). Evaluation of cold stress of young industrial chicory (ci- chorium intybus l.) by chlorophyll a fluorescence imaging. ii. dark relaxation kinetics. Photosynthetica.

[43] Sharif M., Irum I., Yasmin M., Raza M. (2017). Salt & pep- per noise removal from digital color images based on mathematical morphology and fuzzy decision. Nepal J. Sci. Technol..

[44] Irum I., Sharif M., Raza M., Mohsin S. (2015). A nonlinear hybrid filter for salt & pepper noise removal from color images. J. Appl. Res. Technol..

[45] Irum I., Sharif M., Yasmin M., Raza M., Azam F. (2015). A noise adaptive approach to impulse noise detection and reduction. Nepal J. Sci. Technol..

[46] Scharr H., Minervini M., French A.P., Klukas C., Kramer D.M., Liu X., Luengo I., Pape J.-M., Polder G., Vukadinovic D. (2016). Leaf segmentation in plant phenotyping: a collation study. Mach. Vis. Appl..

[47] Nilsback M.-E., Zisserman A. (2010). Delving deeper into the whorl of flower segmentation. Image Vis Comput..

[48] Najjar A., Zagrouba E. (2012). 2012 International Conference on Commu- Nications and Information Technology (ICCIT).

[49] Wang J., He J., Han Y., Ouyang C., Li D. (2013). An adaptive thresholding algorithm of field leaf image. Comput. Electron. Agric..

[50] Prasetyo E., Adityo R.D., Suciati N., Fatichah C. (2017). 2017 3rd International Conference on Science and Technology-Computer (ICST).

[51] Saleem R., Shah J.H., Sharif M., Yasmin M., Yong H.S., Cha J. (2021). Mango leaf disease recognition and classification using novel segmentation and vein pattern technique. Appl. Sci..

[52] Saleem R., Hussain Shah J., Sharif M., Jillani Ansari G. (2021). Mango leaf disease identification using fully resolution convolutional network. Comput. Mater. Continua (CMC).

[53] Das Choudhury S., Goswami S., Bashyam S., Samal A., Awada T. (2017). Proceedings of the IEEE International Conference on Computer Vision Workshops.

[54] Fu L., Tola E., Al-Mallahi A., Li R., Cui Y. (2019). A novel image processing algorithm to separate linearly clustered kiwifruits. Biosyst. Eng..

[55] Abinaya A., Roomi S.M.M. (2016). 2016 International Conference on Communication and Electronics Systems (ICCES).

[56] Aydın D., Uğur A. (2011). Extraction of flower regions in color images using ant colony optimization. Procedia Comput. Sci..

[57] Dubey S.R., Dixit P., Singh N., Gupta J.P. (2013).

[58] Niu X., Wang M., Chen X., Guo S., Zhang H., He D. (2014). Proceedings of the 2014 International Conference on Advanced Mechatronic Systems.

[59] Hinojosa C., Rojas F., Castillo S., Arguello H. (2021). Hyperspectral image segmentation using 3d regularized subspace clustering model. J. Appl. Remote Sens..

[60] Aich S., Ian Stavness (2017). Proceedings of the IEEE International Conference on Computer Vision Workshops.

[61] Grimm J., Herzog K., Rist F., Kicherer A., Töpfer R., Steinhage V. (2019). An adaptable approach to automated visual detection of plant organs with applications in grapevine breeding. Biosyst. Eng..

[62] Itzhaky Y., Guy F., Khoroshevsky F., Shpigler A., Bar-Hillel A. (2018). Leaf counting: Multiple scale regression and detection using deep CNNs. BMVC.

[63] Lu J., Hu J., Zhao G., Mei F., Zhang C. (2017). An in-field automatic wheat disease diagnosis system. Comput. Electron. Agric..

[64] Morris D. (2018). 2018 15th Con- Ference on Computer and Robot Vision (CRV).

[65] Sadeghi-Tehran P., Virlet N., Ampe E.M., Reyns P., Hawkesford M.J. (2019). Deepcount: in-field automatic quantification of wheat spikes using simple linear iterative clustering and deep convolutional neural networks. Front. Plant Sci..

[66] Sapoukhina N., Samiei S., Rasti P., Rousseau D. (2019). Proceedings of the IEEE/CVF Conference on Computer Vision and Pattern Recognition Workshops.

[67] Giuffrida M.V., Scharr H., Tsaftaris S.A. (2017). Proceedings of the IEEE International Conference on Computer Vision Workshops.

[68] Ward D., Moghadam P., Hudson N. (2018). Deep leaf segmentation using synthetic data. arXiv.

[69] Zhu Y., Aoun M., Krijn M. (2018). Joaquin Vanschoren, and High Tech Cam- pus. Data augmentation using conditional generative adversarial networks for leaf counting in arabidopsis plants. BMVC.

[70] Zhang X., Han L., Dong Y., Shi Y., Huang W., Han L., González-Moreno P., Ma H., Ye H., Sobeih T. (2019). A deep learning-based approach for automated yellow rust disease detection from high- resolution hyperspectral uav images. Rem. Sens..

[71] Sapoukhina N., Boureau T., Rousseau D. (2022). Plant disease symptom segmentation in chlorophyll fluorescence imaging with a synthetic dataset. Front. Plant Sci..

[72] Shahzad A., Sharif M., Raza M., Hussain K. (2008). Enhanced watershed image processing segmentation. J. Inf. Commun. Technol..

[73] Pan S., Kudo M., Toyama J. (2009). 2009 First International Conference on Information Science and Engineering.

[74] Nilsback M.-E. (2009).

[75] Thendral R., Suhasini A., Senthil N. (2014). 2014 International Conference on Communication and Signal Processing.

[76] Patel H.N., Jain R.K., Joshi M.V. (2012). Automatic segmentation and yield measurement of fruit using shape analysis. Int. J. Comput. Appl..

[77] Zeng Q., Miao Y., Liu C., Wang S. (2009). Algorithm based on marker-controlled watershed transform for overlapping plant fruit segmentation. Opt. Eng..

[78] Deepa P., Geethalakshmi S.N. (2011). 2011 International Conference on Process Automation, Control and Computing.

[79] Pape J.-M., Klukas C. (2014). European Conference on Computer Vision.

[80] Kumar J.P., Domnic S. (2017). 2017 IEEE International Conference on Current Trends in Advanced Computing (ICCTAC).

[81] Sakurai S., Uchiyama H., Shimada A., Arita D., Taniguchi R. (2018). Two-step transfer learning for semantic plant segmentation. ICPRAM.

[82] M. Trivedi and A. Gupta. Automatic monitoring of the growth of plants using deep learning-based leaf segmentation

[83] Guo R., Qu L., Niu D., Li Z., Yue J. (2021). Proceedings of the IEEE/CVF Interna- Tional Conference on Computer Vision.

[84] Tu Y.-L., Lin W.-Y., Lin Y.-C. (2022). Toward automatic plant phenotyping: starting from leaf counting. Multimed. Tool. Appl..

[85] Al-Shakarji N.M., Kassim Y.M., Palaniappan K. (2017). 2017 IEEE Applied Im- Agery Pattern Recognition Workshop (AIPR).

[86] Xu L., Ye L., Sun Y., Song L., Jin S. (2018). 2018 Joint 10th International Conference on Soft Computing and Intelligent Sys- Tems (SCIS) and 19th International Symposium on Advanced Intelligent Systems (ISIS).

[87] Bell J., Dee H.M. (2019). Leaf segmentation through the classification of edges. arXiv.

[88] Kuznichov D., Alon Z., Honen Y., Kimmel R. (2019). Proceedings of the IEEE/CVF Conference on Computer Vision and Pattern Recognition Work- Shops.

[89] Sabanci K., Kayabasi A., Toktas A. (2017). Computer vision-based method for classification of wheat grains using artificial neural network. J. Sci. Food Agric..

[90] Sabanci K., Toktas A., Kayabasi A. (2017). Grain classifier with computer vision using adaptive neuro-fuzzy inference system. J. Sci. Food Agric..

[91] khan M.A., Akram T., Sharif M., Saba T. (2020). Fruits diseases classification: exploiting a hierarchical framework for deep features fusion and selection. Multimed. Tool. Appl..

[92] Saeed F., Khan M.A., Sharif M., Mittal M., Goyal L.M., Roy S. (2021). Deep neural network features fusion and selection based on PLS regression with an application for crops diseases classification. Appl. Soft Comput..

[93] Lo Bianco M., Grillo O., Escobar Garcia P., Mascia F., Venora G., Bacchetta G. (2017). Morpho-colorimetric characterisation of malva alliance taxa by seed image analysis. Plant Biol..

[94] Kianat J., Khan M.A., Sharif M., Akram T., Rehman A., Saba T. (2021). A joint framework of feature reduction and robust feature selection for cucumber leaf diseases recognition. Optik.

[95] Zhu Q., Zhong Y., Zhao B., Xia G.-S., Zhang L. (2016). Bag-of- visual-words scene classifier with local and global features for high spatial res- olution remote sensing imagery. Geosci. Rem. Sens. Lett. IEEE.

[96] Adeel A., Khan M.A., Sharif M., Azam F., Shah J.H., Umer T., Wan S. (2019). Diagnosis and recognition of grape leaf diseases: An automated system based on a novel saliency approach and canonical correlation analysis based multiple features fusion. Sustain. Comput. Inf. Syst..

[97] Khan M.A., Akram T., Sharif M., Javed K., Raza M., Saba T. (2020). An automated system for cucumber leaf diseased spot detection and classification using improved saliency method and deep features selection. Multimed. Tool. Appl..

[98] Lo Bianco M., Grillo O., Cañadas E., Venora G., Bacchetta G. (2017). Inter-and in- traspecific diversity in cistus l.(cistaceae) seeds, analysed with computer vision techniques. Plant Biol..

[99] Lee S.H., Chan C.S., Mayo S.J., Remagnino P. (2017). How deep learning extracts and learns leaf features for plant classification. Pattern Recogn..

[100] Shah F.A., Khan M.A., Sharif M., Tariq U., Khan A., Kadry S., Thinnukool O. (2021). A cascaded design of best features selection for fruit diseases recognition. Comput. Mater. Continua (CMC).

[101] Khan M.A., Akram T., Sharif M., Alhaisoni M., Saba T., Nawaz N. (2021). A probabilistic segmentation and entropy-rank correlation-based feature selection approach for the recognition of fruit diseases. EURASIP J. Image Video Process..

[102] Piiroinen R., Heiskanen J., Maeda E., Viinikka A., Pellikka P. (2017). Classification of tree species in a diverse african agroforestry landscape using imaging spectroscopy and laser scanning. Rem. Sens..

[103] Koh J.C.O., Spangenberg G., Kant S. (2021). Automated machine learning for high-throughput image-based plant phenotyping. Rem. Sens..

[104] Liu J., Tang Z., Zhang J., Chen Q., Xu P., Liu W. (2016). Visual perception-based statistical modeling of complex grain image for prod- uct quality monitoring and supervision on assembly production line. PLoS One.

[105] Sadeghi-Tehran P., Sabermanesh K., Virlet N., Hawkesford M.J. (2017). Automated method to determine two critical growth stages of wheat: heading and flowering. Front. Plant Sci..

[106] Mohanty S.P., Hughes D.P., Salathé M. (2016). Using deep learning for image-based plant disease detection. Front. Plant Sci..

[107] Brahimi M., Boukhalfa K., Moussaoui A. (2017). Deep learning for tomato diseases: classification and symptoms visualization. Appl. Artif. Intell..

[108] Ghosal S., Blystone D., Singh A.K., Ganapathysubrama- nian B., Singh A., Sarkar S. (2018). An explainable deep machine vision frame- work for plant stress phenotyping. Proc. Natl. Acad. Sci. USA.

[109] Wang G., Sun Y., Wang J. (2017). Automatic Image-Based Plant Disease Severity Estimation Using Deep Learning. Comput. Intell. Neurosci..

[110] Ferentinos K.P. (2018). Deep learning models for plant disease detection and diagnosis. Comput. Electron. Agric..

[111] Ramcharan A., Baranowski K., McCloskey P., Ahmed B., Legg J., Hughes D.P. (2017). Deep learning for image-based cassava disease detection. Front. Plant Sci..

[112] Liu B., Zhang Y., He D.J., Li Y. (2017). Identification of apple leaf diseases based on deep convolutional neural networks. Symmetry.

[113] Picon A., Alvarez-Gila A., Seitz M., Ortiz-Barredo A., Echazarra J., Johannes A. (2019). Deep convolutional neural networks for mobile capture device-based crop disease classification in the wild. Comput. Electron. Agric..

[114] Too E.C., Yujian L., Njuki S., Yingchun L. (2019). A comparative study of fine-tuning deep learning models for plant disease identification. Comput. Electron. Agric..

[115] Amin J., Anjum M.A., Sharif M., Kadry S., Nam Y. (2021).

